# Adipose Derived Mesenchymal Stem Cells Efficiently Rescue Carbon Tetrachloride-Induced Acute Liver Failure in Mouse

**DOI:** 10.1155/2014/103643

**Published:** 2014-06-04

**Authors:** Lisi Deng, Guangze Liu, Xin Wu, Yaping Wang, Minghua Tong, Bing Liu, Kuidong Wang, Yuting Peng, Xiangping Kong

**Affiliations:** ^1^Center of Infectious Diseases, 458th Hospital of PLA, No. 801 Dongfengdong Road, Guangzhou, Guangdong 510600, China; ^2^Southern Medical University, Guangzhou, Guangdong 510515, China; ^3^Institute of Life Science and Bio-Pharmaceutics, Shenyang Pharmaceutical University, Shenyang 110016, China; ^4^Guangzhou Eight People's Hospital, Guangdong 510060, China

## Abstract

*Background and Aim.* Adipose derived mesenchymal stem cells (ADMSCs) may be an attractive source for acute and chronic liver injury because they are abundant and easy to obtain. We aim to investigate the efficacy of ADMSCs transplantation in the acute liver failure (ALF) caused by carbon tetrachloride (CCl_4_) in mice. *Methods.* ADMSCs were isolated from inguinal fat pads of enhanced green fluorescent protein (EGFP) transgenic mice and their surface markers and differentiation potential were analyzed. ALF models were established by infusion of CCl_4_ and divided into two groups: control group; EGFP-ADMSCs transplantation group. The restoration of biological functions of the livers receiving transplantation was assessed via a variety of approaches such as survival rates, live function parameters, histological localization of EGFP-ADMSCs, and Immunofluorescence analysis. *Results.* ADMSCs were positive for CD105, CD44 but negative for CD45, CD34 and had adipogenic, osteogenic differentiation potential. The survival rate of transplantation group significantly increased compared to PBS group. Furthermore, the transplanted cells were well integrated into injured livers and produced albumin, cytokeratin-18. *Conclusion.* Direct transplantation of ADMSCs is an effective treatment for ALF. The transplanted ADMSCs exhibit the potential to differentiate into hepatocyte-like cells in the injured livers.

## 1. Introduction

Acute liver failure (ALF), with a high mortality rate, is a serious clinical condition. Up to now, orthotopic liver transplantation is the only resolutive treatment for ALF and end-stage liver disease [1]. However, for the reason of limited number of donors and organ rejection, alternative approaches are needed. Currently, the development of cell therapy for the treatment of end-stage hepatic diseases is under active investigation [2, 3]. And the cell therapy can be defined as “the use of living cells to restore, maintain, or enhance tissue and organ function” [4]. While the preeminent candidate stem cells for therapy of an injured liver are mesenchymal stem cells (MSCs), MSCs are adherent, fibroblast-like, pluripotent, and nonhematopoietic progenitor cells, which reside in various tissues such as the bone marrow [5], adipose tissue [6], umbilical cords [7], and placenta [8]. Adipose derived mesenchymal stem cells (ADMSCs) are an attractive source for regenerative medicine because they are abundant and easy to obtain from liposuction aspirates or excised fat [9]. They also display multilineage differentiation potential and can differentiate into hepatocyte-like cells* in vitro* [10, 11]. Moreover, ADMSCs are well tolerated and exert* in vitro* and* in vivo* immunoregulatory properties [12]. It has been reported that ADMSCs demonstrate therapeutic efficacy in liver injuries and secrete cytokines and growth factors associated with hepatic regeneration [13–17]. However, the use of ADMSCs to treat acute liver failure in animal models is largely unknown. Additionally, because of difficulty in tracking transplanted ADMSCs-derived hepatocytes in patients, the roles of ADMSCs in liver regeneration have not been fully elucidated. In the current study, we investigated whether the transplantation of ADMSCs is an effective method to prevent ALF in CCl_4_-induced animal model. Furthermore, the homing of ADMSCs to the injured liver and their differentiation into hepatocytes were investigated.

## 2. Materials and Methods

### 2.1. Isolation and Culture of ADMSCs

ADMSCs were isolated from inguinal fat pads of enhanced green fluorescent protein (EGFP) transgenic C57BL/6 mice (Cyagen Biosciences, Guangzhou, China). Adipose tissues were washed extensively with phosphate-buffered saline (PBS) and digested with 0.075% collagenase type I prepared in PBS at 37°C for 60 min with shaking. Enzyme activity was neutralized by adding 5 mL of alpha-MEM containing 10% heat inactivated fetal bovine serum (FBS, Gibco, USA). The solution was filtered through a 100 *μ*m nylon mesh and centrifuged at 1500 rpm for 8 min. The pellet was washed twice with PBS and centrifuged at 1000 rpm for 5 min. The cells were resuspended in stromal medium (alpha-MEM, Gibco, USA) supplemented with 10% FBS and maintained in a humidified tissue culture incubator at 37°C with 5% CO_2_. Upon reaching an 80% confluence, the cells were digested with 0.25% trypsin/EDTA, centrifuged, and resuspended in the same culture medium. After 3 passages, the cells were used for analysis.

### 2.2. Phenotypic Analysis via Flow Cytometry

Phenotypic analyses of cultured ADMSCs were performed prior to transplantation via standard flow cytometry methods. The third and fifth passage cells were harvested, suspended at a concentration of 1 × 10^6^ cells/100 *μ*L in PBS, and incubated with anti-mouse monoclonal antibodies against CD44-phycerythrin (PE), CD105-PE, CD34-allophycocyanin (APC), and CD45-APC (BD Biosciences, San Jose, CA) for 30 min in the dark at 4°C. Cells were washed and analyzed using a FACSCalibur (BD, USA).

### 2.3. Adipogenic and Osteogenic Differentiation

ADMSCs were plated at a density of 2 × 10^3^/well in six-well plates (Corning, USA) and cultured in special conditional adipogenic medium (OriCell MSC Adipogenic Differentiation Medium, Cyagen Biosciences, Guangzhou, China). After 2-3 weeks, adipogenic differentiation was evaluated by Oil red O staining. To induce osteogenic differentiation, ADMSCs were cultured in special conditional osteogenic differentiation medium (OriCell MSC Osteogenic Differentiation Medium, Cyagen Biosciences, Guangzhou, China). After 3 weeks, osteogenic differentiation was evaluated by Alizarin Red staining.

### 2.4. Mice Model and Cell Transplantation

Male C57BL/6 mice weighing 20–23 g were obtained from Guangdong Laboratory Animals (Guangzhou, Guangdong, China). Animal procedures were approved by the Animal Ethics Committee of Southern Medical University. To establish the acute liver failure (ALF) mice model, CCl_4_ (Sigma-Aldrich) was dissolved in olive oil at 20% concentration and injected intraperitoneally into adult male C57BL/6 mice at a dose of 2.8 mL/kg. Cells were transplanted under ethyl-ether inhalation anesthesia at 24 h after administration of CCl_4_. Experimental mice were divided into two groups: transplantation group (*n* = 8), which received EGFP-ADMSCs (5 × 10^6^ cells in 100 *μ*L PBS) via the tail vein; control group (*n* = 6), which receive the equal volume of PBS without cells. After the cell transplantation, blood samples were collected, and the entire livers were taken, fixed, and prepared for further analysis from sacrificed mice.

### 2.5. Long-Term Survival and Biochemical Analysis

Mice that had lived for more than 168 h after transplantation were considered to be survivors. Serum aspartate aminotransferase (AST) and alanine aminotransferase (ALT) levels in mice blood were analyzed prior to ADMSCs transplantation and then on days 7 and 14 after transplantation with an automated biochemical analyzer.

### 2.6. Pathology and Immunofluorescence

Liver tissue samples were harvested at the indicated time points after cell transplantation, fixed in 4% paraformaldehyde, embedded in paraffin, and stained with hematoxylin and eosin (H&E). Frozen sections (5 *μ*m thick) were used for immunofluorescence staining. Sections were blocked with 5% bovine serum albumin in PBS and then incubated with anti-mouse ALB antibody (1 : 200, Abcam) and anti-mouse CK18 antibody (1 : 250, Abcam) according to the manufacturer's instructions. Cy3-conjugated IgG antibody was used as a secondary antibody (1 : 500, Abcam). The sections were observed under a fluorescent microscope (OLYMPUS, BX53F, Japan).

### 2.7. Statistical Analysis

Survival was analyzed using a Kaplan-Meier plot and log-rank analysis. Data were presented as the mean ± SD. Differences in serum levels of biochemical parameters were analyzed using Student's *t*-test with SPSS software version 13.0 (SPSS, Chicago, IL). The significance for all statistical analyses was defined as *P* < 0.05.

## 3. Results

### 3.1. Characteristics of ADMSCs

**Figure 1:**
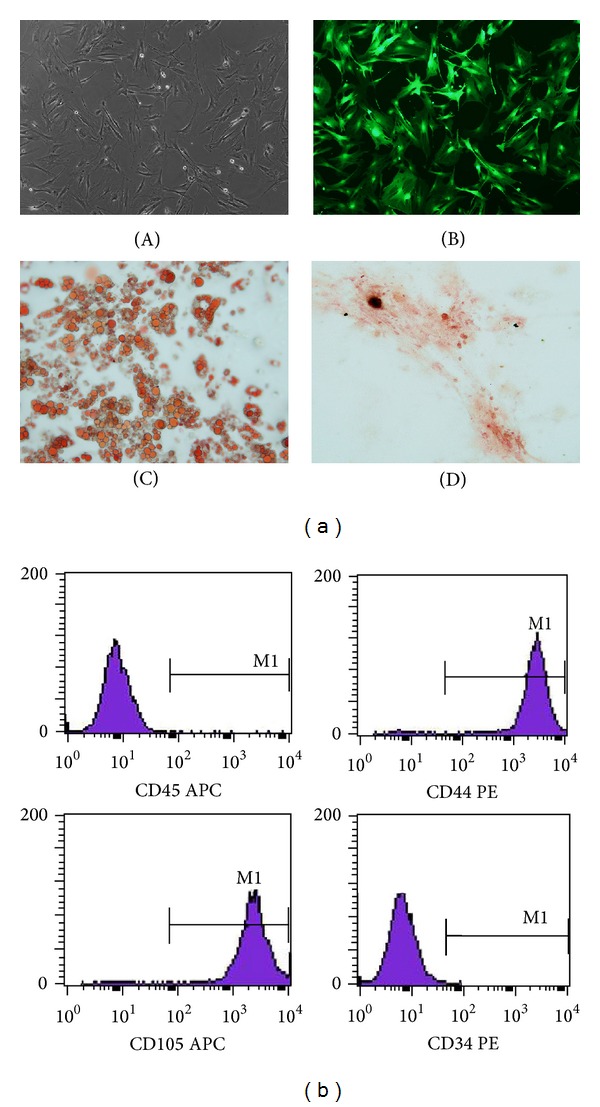
Characteristics of ADMSCs. (a) The morphology of adipose derived mesenchymal stem cells (ADMSCs) and ADMSCs differentiated into adipocytes, osteocytes. ADMSCs exhibited a fibroblast-like morphology on light microscopic images and show green on fluorescence microscopic images (magnification ×10) (A, B). The adipogenic differentiation of the ADMSCs was characterized by Oil red O (magnification ×20) (C). The osteogenic differentiation of ADMSCs was shown by calcium mineralization as revealed by Alizarin Red (magnification ×20) (D). (b) Phenotype profile of ADMSCs determined by flow cytometry.

The morphology of transgenic ADMSCs is consistent with that of normal ADMSCs. EGFP-ADMSCs are fibroblastic in appearance and fluoresce when examined through a FITC filter (Figures 1(A) and 1(B)). The analysis by flow cytometry revealed that these cells were negative for the expression of hematopoietic markers CD45 and CD34, but positive for CD105 and CD44, which are generally considered as markers of MSCs (Figure 1(b)). The multiple differentiation potential of ADMSCs was demonstrated by inducing adipogenic and osteogenic differentiation. The adipogenic differentiation of the ADMSCs was characterized by Oil red O staining, and lipid droplets were visible in the differentiated adipocytes on day 14 after the induction of differentiation (Figure 1(C)). Osteogenic differentiation yielded an extracellular precipitate, which was identified as calcium deposits by the Alizarin Red stain; differentiated ADMSCs formed bone nodules and stained positive (Figure 1(D)). These results indicate that the cells used for transplantation exhibited the classic ADMSC phenotype and multipotential stem cell characteristics, and the presence of EGFP does not seem to change stem cell properties.

### 3.2. Improve Survival Rate and Liver Function by ADMSCs Transplantation

**Figure 2:**
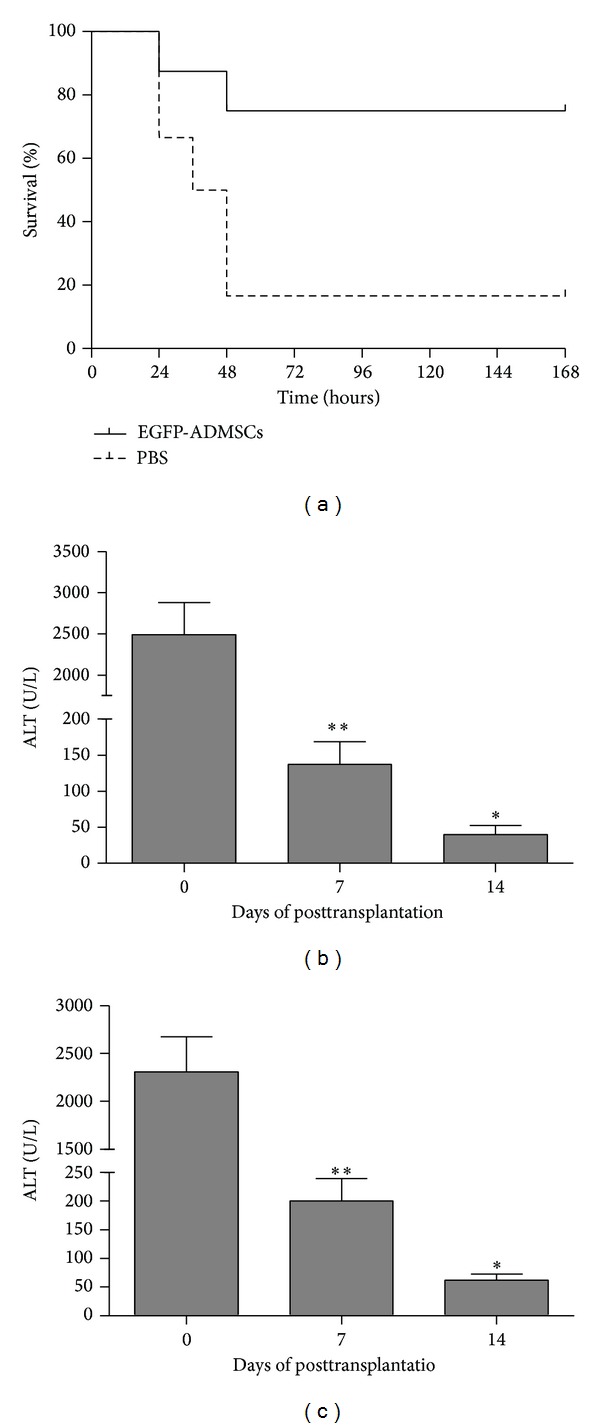
Survival rate and liver function. (a) The survival rate of transplantation group (ADMSCs) was prolonged significantly compared with the control group (PBS) (*P* < 0.05). (b) Alanine aminotransferase (ALT) and (c) aspartate aminotransferase (AST) enzyme release levels in peripheral blood samples collected at pretransplantation and 7 and 14 days after transplantation. **P < 0.01, versus 7 days after transplantation and before transplantation; *P < 0.05, versus 14 days and 7 days after transplantation and before transplantation.

The mice that survived for 7 days (168 h) could usually survive for long term. Five of six animals in a negative control group died between 12 h and 48 h after PBS treatment. However, six of eight animals treated with ADMSCs recovered from ALF. The survival rate in the ADMSCs group was significantly higher than the PBS group (*P* < 0.05) (Figure 2(a)).

As shown in Figures 2(b) and 2(c), the serum ALT and AST levels in the injured mice increased to 2800 and 2600 IU, respectively, at 24 h after administration of CCl_4_, confirming the infliction of acute extensive liver injury by CCl_4_. The mean AST and ALT levels were dramatically decreased in the ADMSCs group at 7 days after transplantation and maintained at normal levels at 14 days (Figures 2(b) and 2(c)). Compared with the control procedures, ADMSC transplantation via the tail vein significantly improved liver function and prevented death from ALF.

### 3.3. Promote Liver Regeneration by ADMSCs Transplantation

**Figure 3:**
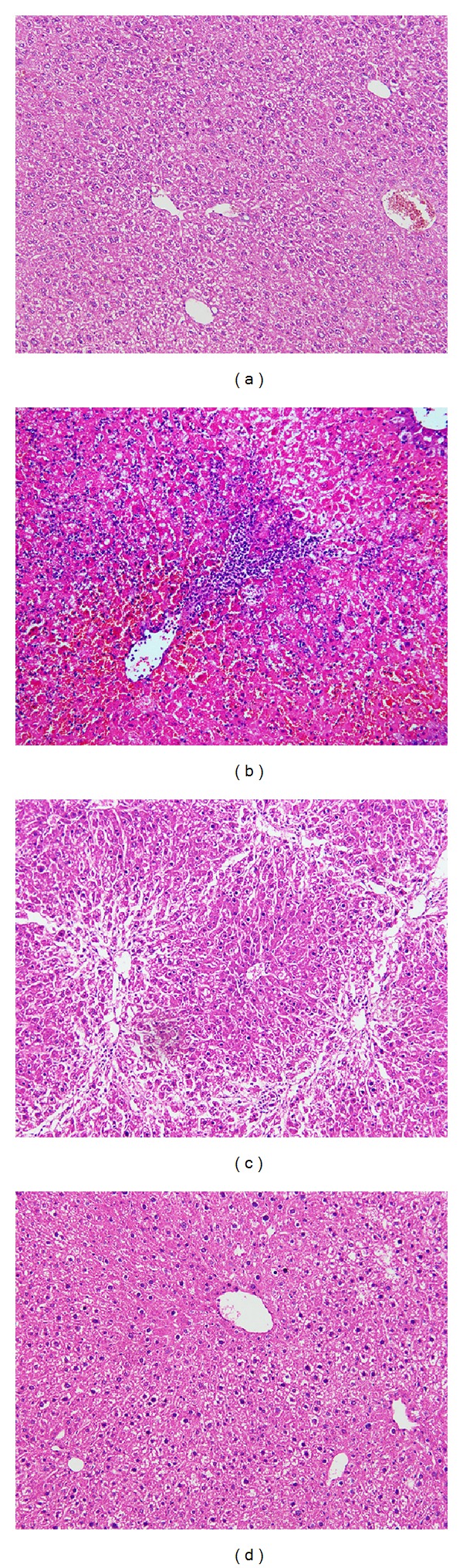
H&E staining. (a) Normal liver tissue. (b) ALF mice showed large areas of inflammation, sinusoid congestion and hemorrhage, and extensive hepatocyte necrosis. (c) New lobules were regenerated at 7 days after infusion of ADMSCs. (d) The impaired hepatic lobule returned to a nearly normal architecture at 28 days after transplantation. Bar = 50 *μ*m.

ALF was validated by the observation of massive necrosis during histological examination. H&E staining (Figure 3(b)) showed large areas of inflammation, sinusoid congestion and hemorrhage, and extensive hepatocyte necrosis. Very few residual hepatocytes were found and the hepatocytes present had a swollen cytoplasm. Transplantation of ADMSCs via the tail vein had a beneficial effect on recovery of the CCl_4_-injured liver. New lobules were regenerated in the surviving animals at 7 days after infusion of ADMSC (Figure 3(c)). Microthrombosis in the sinusoids disappeared and hepatocyte necrosis was suppressed. Furthermore, the impaired hepatic lobule returned to a nearly normal architecture at 28 days after transplantation (Figure 3(d)).

### 3.4. Tracing of Transplanted Cells

**Figure 4:**
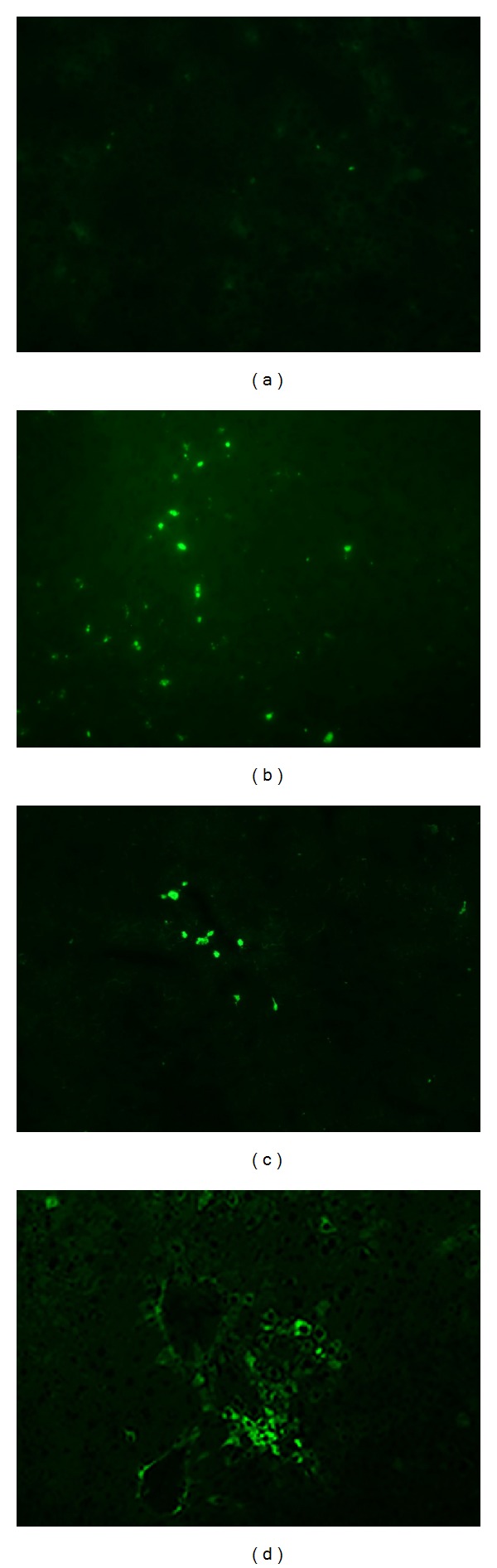
The fluorescent images of EGFP-ADMSCs transplanted mice liver sections. (a) Control group. The EGFP-positive cells were widely distributed in the hepatic lobules on day 7 (b), appearing as small cluster on day 14 (c) and as a mass on day 28 (d) after cell transplantation. Bar = 50 *μ*m.

To investigate whether EGFP-ADMSCs are capable of engrafting in CCl_4_ injured liver, animals were sacrificed 1, 2, and 4 weeks after treatment. In the EGFP-ADMSCs group, the EGFP-positive cells (ADMSCs) were easily detected in the liver by fluorescence microscopy. The transplanted cells appeared in hepatic lobules as scattered individual cells at 7 days (Figure 4(b)), as small cluster at 2 weeks (Figure 4(c)), and as a mass at 4 weeks (Figure 4(d)). The cells showed a hepatocyte-like shape at 4 weeks* in vivo*. This result suggests that the transplanted cells were well integrated in the liver parenchyma. No EGFP-positive cells were observed in the liver specimens of control group (Figure 4(a)).

**Figure 5:**
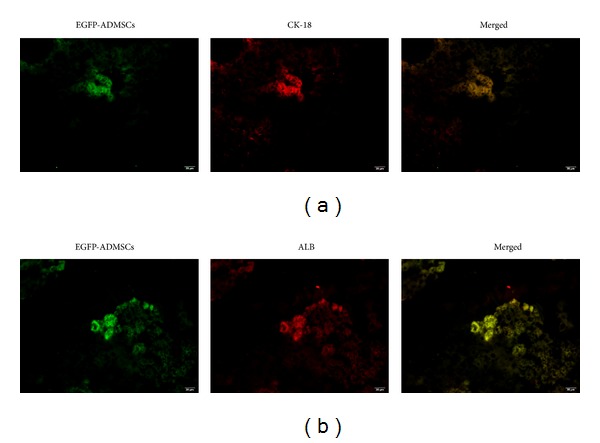
Immunofluorescence of liver sections at 28 days after cell transplantation. The EGFP-positive cell showed a hepatocyte-like shape and expressed CK-18 (a), ALB (b). Bar = 50 *μ*m.

Four weeks after cell transplantation, the transplanted EGFP-positive cells seen in mouse liver sections were found to express cytokeratin-18 (CK-18) (Figure 5(a)) and albumin (ALB) (Figure 5(b)). Thus, the data demonstrate the capability of ADMSCs to differentiate into hepatocyte-like cells and repopulate the liver in ALF.

## 4. Discussion

Several reports have demonstrated the safety and promising beneficial effects of MSCs in the treatment of chronic injury [18–20] and hepatic failure [21–23]. However, the value of ADMSCs in acute liver failure has not been studied as other sources of MSCs even though it has clinical advantages [24]. In this study, we observed that ADMSC transplantation is an effective treatment for ALF. We also showed the hepatocyte differentiation and localization of EGFP-positive cells (ADMSCs) after transplantation.

MSCs from EGFP transgenic mice are similar in their fibroblastic morphology and in differentiation capability to those from nontransgenic mice, the expression of EGFP does not seem to affect the ability to differentiate into different lineages, and cells retain their fluorescence even after undergoing differentiation [25]. Our data revealed that, besides having adipogenic and osteogenic differentiation potential* in vitro*, EGFP-ADMSCs expressed CD44 and CD105 but not hematopoietic cell markers CD45 and CD34. Furthermore, MSCs with a fluorescent marker can be used in experiments utilizing transplantation procedures since they can be easily tracked and identified. Previous study demonstrated that the fluorescently labelled MSCs bound preferentially to injured liver when they were infused into carbon-tetrachloride injured mice [26, 27]. Additionally, MSCs represent an advantageous cell type for allogeneic transplantation since MSCs are immune privileged, therefore reducing the risks of transplant rejection [28]. We transplanted allogenic EGFP-ADMSCs via tail vein and found that the transplanted ADMSCs then progressively migrated into the liver lobules as scattered individual cells. Although there was a decrease in EGFP-positive cells after two weeks, we found that the EGFP-positive cells proliferated and became well integrated into the liver parenchyma after four weeks. Thus, direct transplantation of ADMSCs is a suitable delivery approach for ADMSCs to reach the injury site. And direct transfusion within this damaged environment may result in the proliferation.

To date, only a few clinical studies of bone marrow stem cells transplantation for treatment of hepatic failure have been reported in the literature with limited degrees of success [29–31]. To explore the clinical value of ADMSCs, we established an acute liver failure model by CCl_4_ administration. Liver of ALF in animals model undergoes massive necrosis with hemorrhages involving entire lobules, which results in death within 3 days following the induction. In our study, it was found that transplantation of as few as 5 × 10^6^ ADMSCs effectively rescued 80% of the recipient mice from ALF. The recipient mortality was apparently reduced by ADMSC treatment compared with the PBS control group. Additionally, the concentrations of ALT and AST, which are two important indicators of liver injury, were significantly decreased after ADMSCs transplantation. Histopathological evaluation of liver tissue after cell transplantation provided initial insights into the cellular targets of therapy. A striking recovery of liver structure was seen after transplantation, suggesting that ADMSCs may reconstitute liver tissue by either differentiating into hepatocytes or promoting endogenous host regeneration. These results confirmed that the direct transplantation of native ADMSCs can rescue ALF and repopulate livers of mice as an efficient alternative source.

It has been suggested that liver regeneration can occur via the transdifferentiation of the transplanted cells that reside in the regenerating tissue or cell-cell fusion [32–35]. Recently, Li et al. demonstrated that immediate intraportal transplantation of human bone marrow mesenchymal stem cells (hBMSCs) may transdifferentiate into hepatocytes during the initial stage of fulminant hepatic failure (FHF) [32]. However, other reports demonstrated that hepatogenic differentiation after transplantation of BMSCs occurred at a low frequency in a model of acute liver injury [18, 36]. In this study, in liver sections EGFP-ADMSCs presented a liver epithelial morphology and expressed hepatocyte-specific markers as CK-18 and ALB after four weeks of administration of ADMSCs in CCl_4_-treated mice. However, we could not unequivocally prove that the transplanted ADMSCs fully differentiated into cells with functions comparable to endogenous hepatocytes and rescue the ALF animals during the initial 3 days. Kuo et al. demonstrated that the superior rescuing potential of MSCs over MDHs (mesenchymal stem cell-derived hepatocytes) as well as the ability to rescue FHF in such a short period suggested that differentiation of donor cells into hepatocytes was not the primary attribute [37]. The transplanted ADMSCs may participate in liver regeneration via proliferation and stimulate the regeneration of endogenous hepatocytes via secreted molecules rather than protected cells from necrosis.

In conclusion, our study demonstrated that ADMSCs transplantation is effective in treating CCl_4_-induced acute liver failure. ADMSCs are involved in liver regeneration* in vivo*; however, the mechanisms such as paracrine, anti-inflammatory, and antiapoptotic effects of transplanted ADMSCs remain to be resolved. In addition, although ADMSCs are an outstanding source for transplantation and offer a novel therapy for acute liver diseases, it remains to be seen whether ADMSCs transplantation will be effective for treatment of chronic liver diseases of different etiologies.

## Abbreviations

ADMSCs: Adipose derived mesenchymal stem cells
ALF: Acute liver failure
CCl_4_: Carbon tetrachloride
EGFP: Enhanced green fluorescent protein.
